# piR_015520 Belongs to Piwi-Associated RNAs Regulates Expression of the Human Melatonin Receptor 1A Gene

**DOI:** 10.1371/journal.pone.0022727

**Published:** 2011-07-26

**Authors:** Teresa Esposito, Sara Magliocca, Daniela Formicola, Fernando Gianfrancesco

**Affiliations:** Institute of Genetics and Biophysics Adriano Buzzati-Traverso, National Research Council of Italy, Naples, Italy; Beckman Research Institute of the City of Hope, United States of America

## Abstract

Piwi-associated RNAs (piRNAs) are a distinct class of 24- to 30-nucleotide-long RNAs produced by a Dicer-independent mechanism, and are associated with Piwi-class Argonaute proteins. In contrast to the several hundred species of microRNAs (miRNAs) identified thus far, piRNAs consist of more than 30,000 different species in humans. Studies in flies, fish and mice implicate these piRNAs in regulating germ line development, the silencing of selfish DNA elements, and maintaining germ line DNA integrity. Most piRNAs map to unique sites in the human genome, including intergenic, intronic, and exonic sequences. However, the role of piRNAs in humans remains to be elucidated. Here, we uncover an unexpected function of the piRNA pathway in humans. We show for the first time, that the piRNA_015520, located in intron 1 of the human Melatonin receptor 1A (*MTNR1A*) gene, is expressed in adult human tissues (testes and brain) and in the human cell line HEK 293. Although the role of piR_015520 expression in brain tissue remains unknown, the testes-specific expression is consistent with previous findings in several species.

Surprisingly, in contrast to the mechanism known for miRNA-mediated modulation of gene expression, piRNA_015520 negatively regulates *MTNR1A* gene expression by binding to its genomic region. This finding suggests that changes in individual piRNA levels could influence both autoregulatory gene expression and the expression of the gene in which the piRNA is located. These findings offer a new perspective for piRNAs functioning as gene regulators in humans.

## Introduction

Small non-coding RNAs have emerged as potent regulators of gene expression at both the transcriptional and post-transcriptional levels [1]–[3]. Recently, small RNAs that interact with Piwi proteins have been discovered in the mammalian germ line and in Drosophila. These Piwi-interacting RNAs (piRNAs) represent a distinct small RNA pathway and differ from miRNAs in several ways [4]. In flies, piRNA mutations lead to the overexpression and mobilisation of retrotransposons, which results in DNA lesions that cause germ line DNA damage [5]–[9]. The biogenesis and mechanism of action of piRNAs is not well understood. For example, it is not known whether piRNAs primarily control chromatin organisation, gene transcription, RNA stability or RNA translation. Moreover, proteins involved in piRNA production have been implicated in the control of gene expression in somatic cells and in learning and memory [10]. These data suggest that piRNAs might impact a broad range of biological processes.

Studies in mice showed that piRNA-encoding regions are distributed over most chromosomes and range in size from 0.9 to 127 kb. Although piRNAs map exclusively to one chromosomal strand in many regions, some regions encode piRNAs in both orientations. In mammals, piRNAs predominantly map to a single genomic locus, whereas in flies they map to repetitive sites such as transposable elements. Betel et al. describes that 25% of piRNA clusters have 5′ and 3′ ends that coincide, indicating that they are not random degradation products of long transcripts [11]. Because no stem and loop regions have been identified for piRNAs, it is possible that long-range dsRNA structure or sequence-specific protein machinery is involved in guiding the maturation process [12], [13].

In a recent study, we identified a genetic link between variants of intron 1 of the melatonin receptor 1A (*MTNR1A)* gene and calcium nephrolithiasis [14]. In this study we conducted a bioinformatic analysis of this 22 kb genomic region in order to identify possible regulatory elements. From this analysis, we identified the piR_015520-encoding region in intron 1 of the *MTNR1A* gene. Interestingly we demonstrate that the piRNA gene is expressed in human tissues and we show that this small RNA molecule is able to repress the expression of the melatonin receptor 1A gene.

## Results and Discussion

### Bioinformatics analysis and genotyping

The human *MTNR1A* gene is located on the chromosomal region 4q35.2, it spans a genomic region of 21.913 bases and is split in two exons.

The 21.913 bases were interrogated with repeat-masker (http://www.repeatmasker.org/cgi-bin/WEBRepeatMasker) to eliminate Alu and Line repetitive elements, and then the output sequence was targeted against both the NCBI non redundant (NR) and NT databases using BLASTN as tool (http://blast.ncbi.nlm.nih.gov/Blast.cgi). We identified a piRNA-encoding region, a new class of small RNA molecules (PIWI RNA, hsa_piR_015520) in intron 1 of the MTNR1A gene, occurring 14 kb downstream of the ATG start codon.

**Figure 1:**
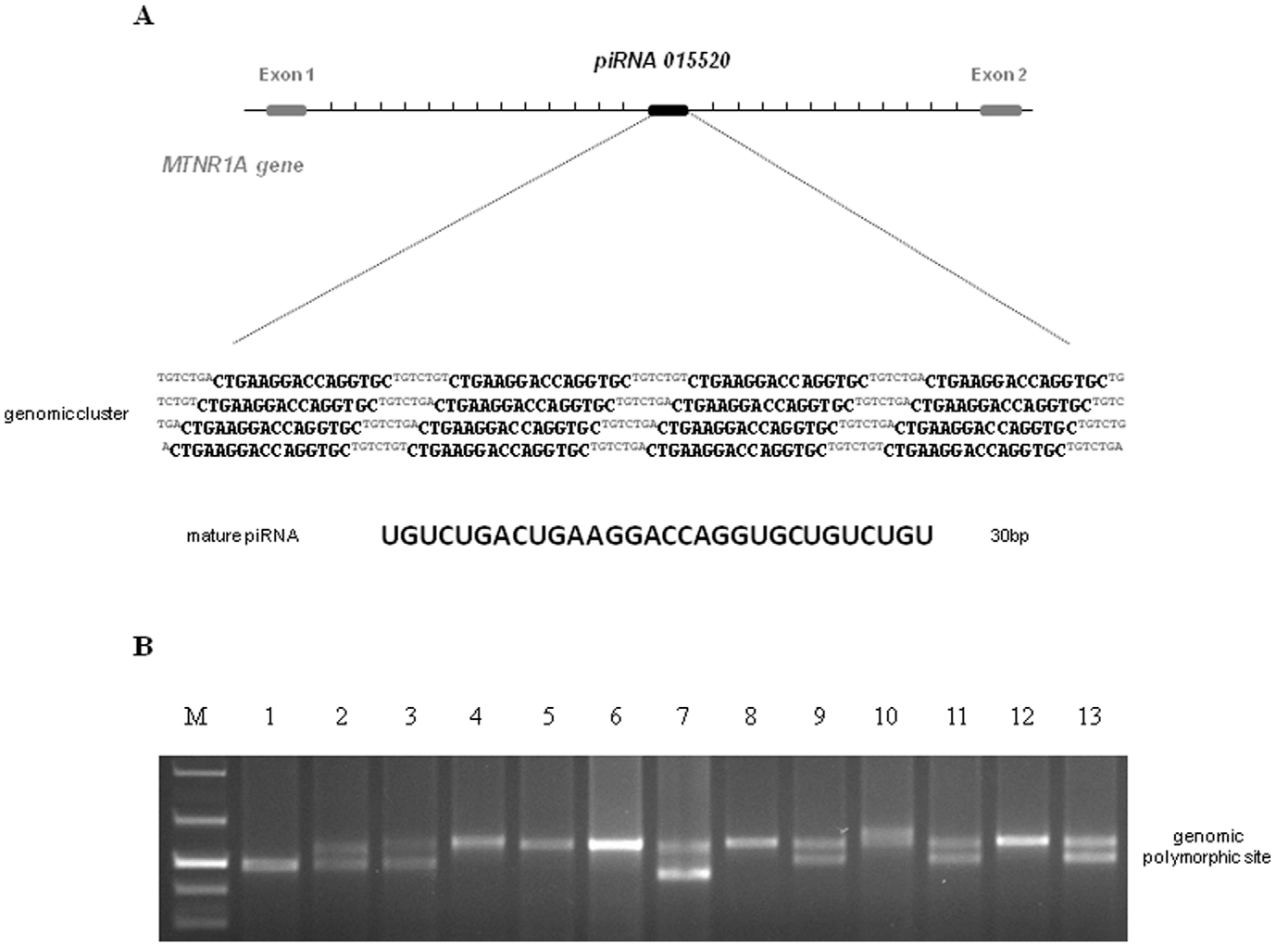
Genomic structure of piRNA_015520. **a,** Physical location and genomic structure of the *MTNR1A* gene and the piRNA_015520. The 30 bp sequence of the mature piRNA is shown, with the directed repeat segments of 7 bp at the 5′ and 3′ end depicted in superscript. **b**, Genotyping of the piRNA_015520 genomic region from Caucasian DNA samples.

Transcripts encoded by piR_015520 belong to a newly identified class of small RNA molecules called piRNAs. The piR_015520 locus is organised in a cluster of 30 bp units and contains directed and overlapping repeat segments of 7 bp at the 5′ and 3′ ends (Fig. 1a). To determine if the piR_015520 genomic region is polymorphic in humans, we designed primers outside the genomic cluster and amplified DNA from 100 unrelated subjects. At least four different bands were detected and sequenced, showing that the genomic cluster could contain 12, 14, 16, and 18 repeats (Fig. 1b). Although our previous results indicate an association between allelic variants of MTNR1A and recurrent calcium nephrolithiasis, we confirm here that this association is not driven by the piR_015520 polymorphism.

### piRNA expression profile

**Figure 2:**
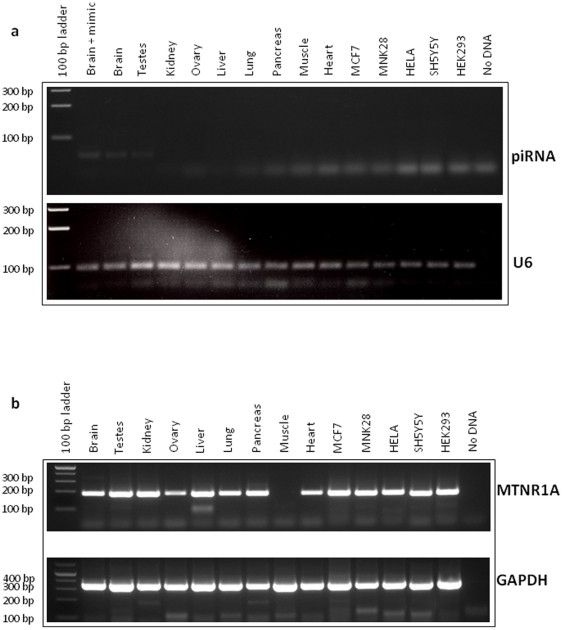
Expression profiling of the piRNA_015520 and *MTNR1A* genes in human adult tissues and cell lines. **a**, 10 ng of total RNA derived from human adult tissues (lanes 2–11) and human cell lines (lanes 12–16) was reverse transcribed with the TaqMan® MicroRNA Reverse Transcription Kit (Applied Biosystems) using piRNA_015520-specific and U6 Reverse Transcription primers (Applied Biosystems). Lane 2 contains the positive control that was made using 10 ng of human brain RNA and 5×10^10^ copies of the synthetic mimic piRNA_015520. qPCR reactions were performed using the TaqMan® Small RNA Assay following the manufacturer's instructions (Applied Biosystems). Briefly, the 20 µl reaction mixture contained 1 ng of cDNA, 1x TaqMan® Small RNA Assay mixture (small RNA-specific forward PCR primer, specific reverse PCR primer and small RNA-specific TaqMan® MGB probe), and 1x TaqMan® Universal PCR Master Mix II (Applied Biosystems). Thermal cycling was performed using the LightCycler System DNA Engine Opticon 2 (MJ Research) with an initial denaturation of 10 min. at 95°C, followed by 50 cycles of 15 sec. at 95°C, 60 sec. at 60°C. Each reaction was performed in triplicate and PCR products were analysed by electrophoresis on 3% agarose gels. The expression profiles were normalised to *U6* mRNA levels to account for differences in starting material and cDNA reaction efficiency. Molecular weight markers (M) and the control lane (no RNA) are shown. **b**, 1 µg of total RNA was reverse transcribed with the Transcriptor HiFi cDNA Synthesis kit (Roche). qPCRs were performed using 10 ng of cDNA, 0.5 µM of human *MTNR1A* gene-specific primers and 1x of FastStart Universal SYBR green Master Mix (Roche) in a total volume of 20 µl. *GAPDH* RNA was used as control.

Two real-time PCR-based piRNA expression assays were designed to detect the specific expression of the mature form of piR_015520 in adult human brain and testes (Fig. 2a).

miRNA can be localised in the intronic regions of host genes. Interestingly, it has been demonstrated that many intronic miRNAs and their host genes are co-regulated and co-transcribed from a common promoter [15]–[17]. To explore the possibility that expression of piR_015520 was related to the host gene, we determined the expression profile of the *MTNR1A* gene using real-time PCR and the same RNA panel used for piRNA expression analysis. Significant expression of the *MTNR1A* gene was found in several tissues. Further, *MTNR1A* exhibited a different expression pattern compared to piR_015520, suggesting that these two genes use different promoter regions (Fig. 2b).

Many miRNAs are aberrantly expressed in various pathologies including cancer and regulate tumor- and metastasis-associated genes [18]. We therefore investigated the expression of piR_015520 in various cell lines: human breast adenocarcinoma cell line (MCF7), human gastric cancer cell line (MNK-28), human epithelial carcinoma cell line (HeLa), human neuroblastoma cell line (SH5Y5Y) and human embryonic kidney cell line (HEK 293). In particular, piR_015520 showed a very low level of expression only in the HEK 293 cell line (CT = 48, undetectable on agarose gel, Fig. 2a).

### *MTNR1A* gene expression regulation

In Drosophila, piRNAs could silence gene expression by promoting heterochromatin assembly, which could directly suppress transcription [19]–[20]. We performed transfection experiments to test the ability of piRNA-015520 to bind its genomic region, thereby affecting gene regulation of the *MTNR1A* host gene.

**Figure 3:**
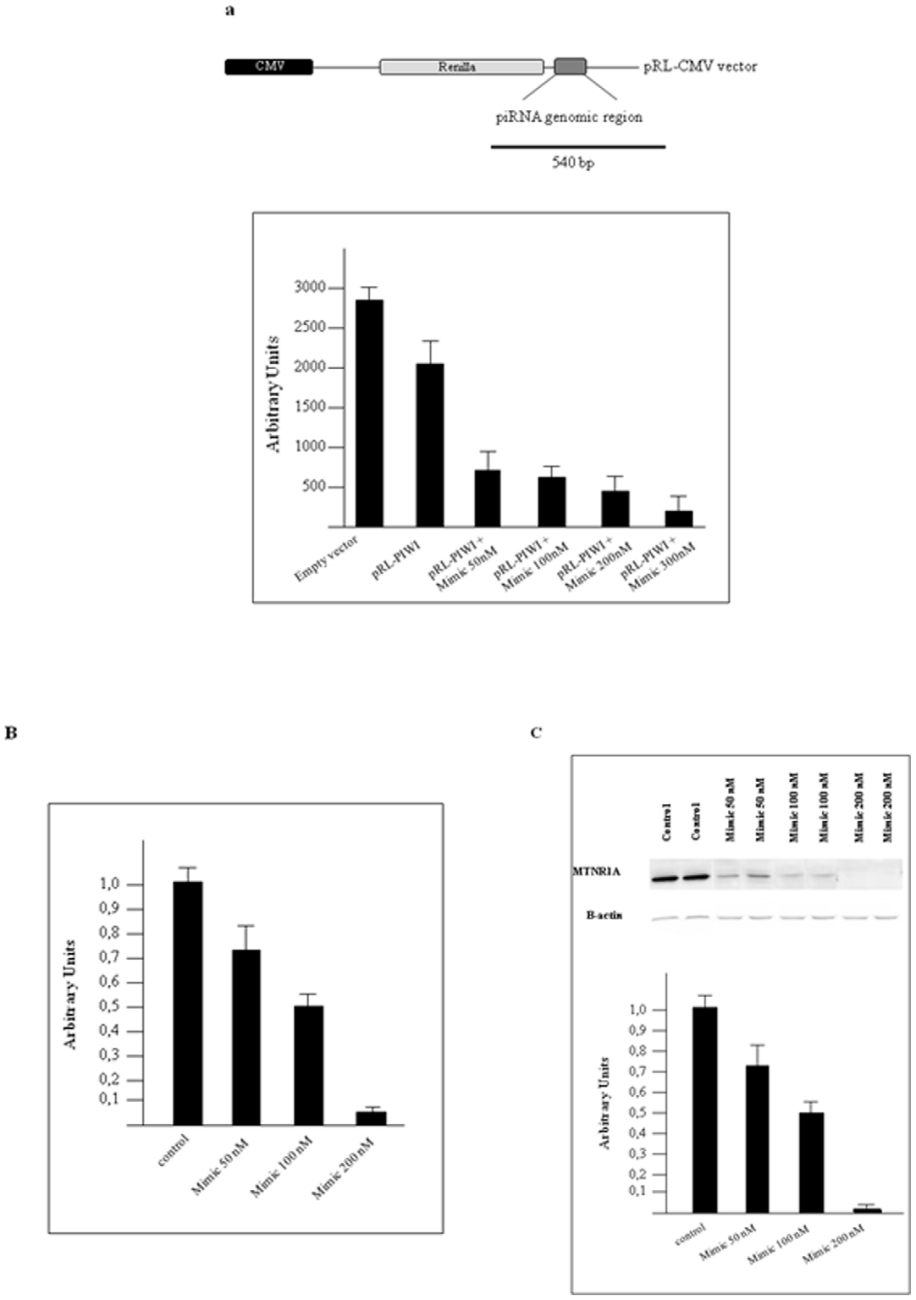
The *MTNR1A* gene is downregulated by a piRNA transcript. **a**, The 500 bp piRNA genomic cluster was ligated into a linearised XbaI pRL-CMV vector (Promega) at the 3′ end of the Renilla luciferase reporter gene; this plasmid is reported as pRL-PIWI. The histogram shows the renilla/luciferase expression ratio after transfection of HEK 293 cells with increasing concentrations of the pRL-PIWI plasmid and the chemically synthesised piRNA mimic (50 nM, 100 nM, 200 nM, 300 nM). **b**, The histogram shows the levels of *MTNR1A* mRNA in HEK 293 cells after transfection with increasing concentrations of the piRNA mimic (50 nM, 100 nM, 200 nM). As controls of transfection we used HEK 293 cells without piRNA mimic and HEK 293 cells trasfected with 200 nM of a dsRNA 30 bp oligo not related with the *MTNR1A* gene. The bar reported as control is a mean value of these two experiments. *GAPDH* mRNA was used as a control. The ratio of *MTNR1A* mRNA/*GAPDH* mRNA was set to 1 in the control (no piRNA mimic transfection). Quantitation of expression levels was determined by RT-qPCR. **c**, Western blots of HEK 293 cells transfected with the piRNA mimic in increasing concentrations (50 nM, 100 nM, 200 nM) were probed with an anti-MTNR1A antibody. Beta-actin was used as a loading control. The histogram shows the ratio of MTNR1A/beta-actin. The ratio was normalised to 1 in the control transfection (as described above). The mean value of three quantitations is shown; error bars correspond to s.d.

The piRNA genomic cluster, which spans about 500 bp and includes 16 repeats, was amplified from genomic DNA and ligated into a linearised pRL-CMV vector (Promega) at the 3′ end of the Renilla luciferase reporter gene. The pRL-PIWI plasmid was transfected into HEK 293 cells, along with increasing concentrations (50 nM, 100 nM, 200 nM, 300 nM) of a chemically synthesised piRNA-015520 mimic, which mimics the function of the same piRNA. Addition of the piRNA mimic was able to promote the repression of Renilla luciferase activity in a concentration-dependent manner (Fig. 3a). To test the endogenous regulation of the *MTNR1A* gene by the piRNA transcript, we transfected HEK 293 cells with increasing concentrations (50 nM, 100 nM, 200 nM) of the piRNA mimic and tested the expression levels of the endogenous *MTNR1A* gene using real-time PCR. We observed a repression of melatonin receptor 1A gene expression in a concentration-dependent manner (Fig. 3b).

These data demonstrate that there is an effective, specific, and functional interaction between a piRNA and its genomic region, suggesting that changes in piRNA levels may effectively modulate its expression and the expression of the gene in which is located.

Repression of the *MTNR1A* gene by increasing concentrations of piRNA transcript was also confirmed by Western blot analysis using an anti-MTNR1A antibody (Novus Biological) (Fig. 3c).

### piRNA-RNA protein interaction

Mature piRNAs are double stranded RNAs of 26–31 nucleotides that form RNA-protein complexes through interactions with Piwi proteins. To demonstrate an interaction between piR_015520 and RNA-binding proteins, we performed an electrophoretic mobility shift assay (EMSA) using a radiolabelled piRNA mimic probe.

**Figure 4:**
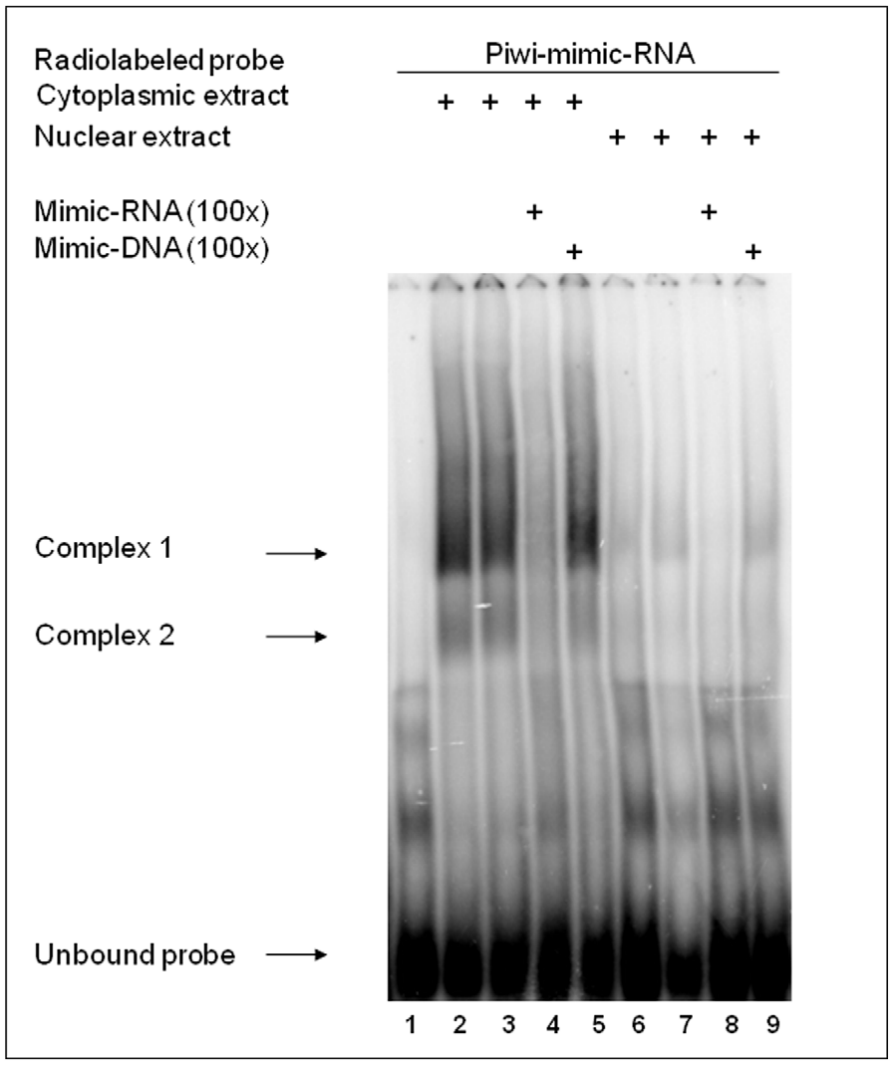
Electrophoretic mobility shift assays (EMSAs). Two distinct complexes (indicated with arrows) formed upon incubation of the piRNA mimic probe with cytoplasmic extracts from HEK 293 cells (lanes 2–5). Low-intensity bands were revealed upon incubation of the piRNA mimic probe with nuclear extracts (lanes 6–9). In competition experiments, a 100-fold molar excess of cold mimic-RNA (lanes 4 and 8) was able to fully compete with the probe, whereas a 30 bp dsDNA could not (lanes 5 and 9), demonstrating that the binding was specific to RNA-binding proteins.

Two distinct complexes were revealed upon incubation of the piRNA mimic probe with cytoplasmic extract from unstimulated HEK 293 cells (Fig. 4). The binding specificity was demonstrated in a competition assay using a 100-fold excess of unlabelled probe. The unlabelled RNA probe was able to fully compete with the probe, whereas the 30 bp dsDNA probe was not, demonstrating that the binding was specific to RNA-binding proteins. Moreover, to determine if the Piwi protein was one of the factors bound to the complexes, an anti-Piwi antibody was used for a supershift assay. No supershift was detected upon addition of the Piwi antibody, suggesting that other RNA-binding proteins not yet identified could bind the piRNA. In conclusion, we have identified a piRNA-encoding region (piR_015520) in the first intron of the human melatonin receptor 1A (*MTNR1A*) gene. We demonstrate that the human gene encoding the small RNA molecule is expressed in human tissues, specifically in brain and testes. To our knowledge, this is the first demonstration that a human piRNA is expressed in tissues distinct from the testes. Interestingly, we detected piRNA expression in the brain where deregulation of the MT1 and MT2 receptors occurs in neurodegenerative diseases such as Alzheimer's disease. Specifically, reduced levels of the MT2 receptor-subtype and enhanced MT1 receptor expression have been described [21]. Further, elevated expression of the MT1 receptor was found in malignant human breast epithelia compared to normal breast epithelia and stroma [22]–[23].

To date, we do not know whether our data for piR_015520 represents a single case or a more general phenomenon. However, if this is also true for a different piRNA, then it is important to take it into account. In fact, piRNAs consist of more than 30,000 different species, in contrast to only several hundred species of miRNAs [24] . Most piRNAs map to the genome in clusters of 20 to 90 kbs in a strand-specific manner, with each cluster likely representing a long single-stranded RNA precursor, or more often, two non-overlapping and divergently transcribed precursors [12]–[13]. Our EMSA shows that the piRNA is able to interact with RNA-binding proteins present in both the nucleus and cytoplasm. However, mouse and Drosophila Piwi-class Argonaute proteins are also present in the cytoplasm, and piRNA–Piwi-class-Argonaute complexes could silence gene expression by targeting the destruction of mature mRNA following exit from the nucleus. It is also possible that piRNA-Argonaute complexes function in both the nucleus and cytoplasm during the development of complex multicellular organisms.

Thus, piRNAs may have a strong impact on gene expression, by affecting epigenetic programming, transposition, and post-transcriptional regulation. This study therefore offers a new perspective for piRNAs functioning as gene regulators, and suggests a role for piRNAs in regulating physiological and/or pathological conditions.

## Materials and Methods

### Ethical approval

This research was reviewed and approved by the University of Naples Human Research Ethics Committee and all subjects participating in the study gave written informed consent.

### Genotyping

Genomic DNA was isolated from whole blood by a standard salting out procedure. The piRNA genomic region genotyped in this study was amplified using the following procedure: an initial denaturation step of 180 sec. at 94°C, followed by 35 cycles of 30 sec. at 94°C, 30 sec. at melting temperature 58°C, 30 sec. at 72°C, and a terminal extension of 10 min. at 72°C.

Primer sequences used were the following: the sense primer sequence was 5′- CCCTTAGTACTTTGCAGCAA -3′, and the antisense sequence was 5′- TCTGTTTGATGCTGTGATGG -3′. PCR products 450-600 bp in length were analysed by electrophoresis on 1.5% agarose gels.

Samples were ExoSap-digested (Amersham) and sequenced using the Big Dye Terminator Ready Reaction Kit (Applied Biosystems). Sequencing reactions were performed on a 9700 Thermal Cycler (Applied Biosystems) for 25 cycles of 95°C for 10 sec., TM for 5 sec. and 60°C for 2 min. After the sequencing, each reaction was column-purified (Amersham) to remove excess dye terminators, and was subsequently run on the ABI prism 3700 Genetic Analyser (Applied Biosystems). Sequences were analysed using multiple alignments of sequences with the program Autoassembler (Applied Biosystems).

### Expression profile of piR_015520

Total RNAs from human adult tissues were purchased from Stratagene. Two different assays were used to show the expression profile of the piR_015520 transcript. In the first assay, 1 µg of total RNA was reverse transcribed with the miScript Reverse Transcription Kit according to the manufacturer's instructions (Qiagen). During the reverse transcription step, miRNAs were polyadenylated with poly(A) polymerase. Reverse transcriptase was used to convert RNA (including precursor miRNA, mature miRNA, other small noncoding RNA, and mRNA) to cDNA using both oligo-dT and random primers. qPCR reactions were performed in triplicate using an oligo-dT primer with a universal tag sequence on the 5′ end, together with the piRNA-specific primer. qPCR reactions were prepared using the miScript SYBR Green PCR Kit (Qiagen) following the manufacturer's directions.

The second assay used for expression profiling was performed with the Custom TaqMan® Small RNA Assays kit (Applied Biosystems). Procedures are reported in the legend for Figure 2. The Figure 2 legend also reports the procedures used to determine *MTNR1A* gene expression levels.

### Oligonucleotides and plasmids

A chemically modified double-stranded piRNA mimic (hsa_piR_015520) was purchased from Qiagen. The sequence of the piRNA is 5′-UGUCUGACUGAAGGACCAGGUGCUGUCUGU-3′. The pRL-CMV plasmid coding for Renilla luciferase (Promega) was modified by insertion of the piRNA genomic sequence in the XbaI restriction site. The direction of the inserted 3′ UTR region was confirmed by PCR and sequencing after ligation. The pGL3 plasmid encoding for firefly luciferase was used as a control.

### Protein extraction and Western blot analysis

For Western blotting, cells were solubilised in lysis buffer (50 mM HEPES pH 7.5, 150 mM NaCl, 4 mM EDTA, 10 mM Na_4_PO_7_, 2 mM Na_3_VO_4_, 100 mM NaF, 10% glycerol, 1% Triton X-100, 1 mM phenylmethylsulfonyl fluoride, 100 mg/mL aprotinin, 1 mM leupeptin) for 60 min. at 4°C. Cell lysates were clarified at 5,000 g for 15 min. Solubilised proteins were then separated by SDS-PAGE and transferred onto Immobilon-P membranes (0.45 µ pore size; Millipore, Bedford, MA). The membrane was probed with a 1∶500 dilution of primary antibody (anti-MTNR1A antibody) and subsequently probed with an anti-goat HRP-conjugated secondary antibody at a 1∶2,000 dilution. Signals were visualised using the Bio-Rad Chemidoc System. Densitometry analysis was performed using Scion Image (Ver. 4.0.2; Scion Corporation, USA). The signals obtained for each protein were normalised to beta-actin, and the mean±SE of three independent experiments was plotted.

All antibodies were purchased from Novus Europe.

Immunoreaction signals were visualised with enhanced chemiluminescence (ECL Plus, Amersham Biosciences).

### Cell culture

Human Embryonic Kidney 293 cells (HEK 293, ATCC CRL-1573) were maintained in Dulbecco's modified Eagle's medium (DMEM) supplemented with 10% fetal bovine serum (GIBCO) and glutamine.

### Transfection and inhibition experiments

The day before transfection, HEK 293 cells were seeded in 24-well plates with 500 µl of antibiotic-free medium and were grown to 70–90% confluence. The standard co-transfection mix was prepared for triplicate samples by adding 75 ng of the pGL3 control plasmid, 30 ng pRL-PIWI (for details see legend to Fig. 3), and with increasing concentrations of the piR_015520 mimic (50 nM, 100 nM, 200 nM, 300 nM) in 150 µl of serum-free DMEM; 3 µl of TransFast reagent (Promega) was added separately in 150 µl serum-free DMEM. The two solutions were mixed, incubated at room temperature for 20-30 min, and 100 µl of the mixture was then added to each well. The final volume of the medium plus the transfection mixture was 600 µl. Cells were incubated with the transfection mixture for 4 hrs.; the medium was then replaced with new fully-supplemented culturing medium. Twenty-four hours after transfection, firefly and Renilla luciferase activities were measured using a dual luciferase assay according to the manufacturer's instructions (Promega).

### Electrophoretic mobility shift assays (EMSAs)

EMSAs were performed using a radiolabelled piRNA mimic probe. Nuclear and cytoplasmic extracts from HEK 293 cells were prepared as described by Granelli-Piperno et al. [25]. Binding reactions (20 µl) contained ^32^P-labeled mimic probe (10^5^ cpm), 10 µg of cytoplasmic extracts (lanes 2–5) or 10 µg of nuclear extracts (lanes 6–9), 2 µg poly dI-dC, 100 mM KCl, 10 mM MgCl_2_, 20 mM HEPES pH 7.9, 0.2 mM EDTA, 0.5 mM dithiothreitol, 20% glycerol, and protease inhibitors. Reaction mixtures were incubated for 30 min. at room temperature, resolved on non-denaturing 5% polyacrylamide gels, dried, and then exposed to autoradiography.
